# Molecular Characterization of Novel *Cryptosporidium* Fish Genotypes in Edible Marine Fish

**DOI:** 10.3390/microorganisms8122014

**Published:** 2020-12-16

**Authors:** Gabriela Certad, Alireza Zahedi, Nausicaa Gantois, Manasi Sawant, Colette Creusy, Erika Duval, Sadia Benamrouz-Vanneste, Una Ryan, Eric Viscogliosi

**Affiliations:** 1 Institut Pasteur de Lille, U1019–UMR 9017–CIIL–Centre d’Infection et d’Immunité de Lille, Université de Lille, CNRS, Inserm, CHU Lille, F-59000 Lille, France; nausicaa.gantois@pasteur-lille.fr (N.G.); manasi.sawant@pasteur-lille.fr (M.S.); sadia.benamrouz@univ-catholille.fr (S.B.-V.); eric.viscogliosi@pasteur-lille.fr (E.V.); 2 Délégation à la Recherche Clinique et à l’Innovation, Groupement des Hôpitaux de l’Institut Catholique de Lille, F-59462 Lomme, France; 3 Harry Butler Institute, Murdoch University, Perth 6150, Australia; A.ZahediAbdi@murdoch.edu.au (A.Z.); Una.Ryan@murdoch.edu.au (U.R.); 4 Service d’Anatomie et de Cytologie Pathologiques, Groupement des Hôpitaux de l’Institut Catholique de Lille (GHICL), F-59000 Lille, France; creusy.colette@ghicl.net (C.C.); duval.erika@ghicl.net (E.D.); 5 Laboratoire Ecologie et Biodiversité, Institut Catholique de Lille, Faculté de Gestion Economie et Sciences, F-59000 Lille, France

**Keywords:** piscine *Cryptosporidium*, edible marine fish, genetic characterization, 18S rDNA gene, actin gene, molecular phylogeny

## Abstract

Current knowledge of *Cryptosporidium* species/genotypes in marine fish is limited. Following phylogenetic analysis at the 18S rDNA locus, a recent study identified six new genotypes of *Cryptosporidium* colonizing edible fish found in European seas. Of these, five grouped in a clade together (#Cryptofish 1–5) and one grouped separately (#Cryptofish 7). In the present study, after phylogenetic analyses of #Cryptofish1, #Cryptofish2, #Cryptofish4, #Cryptofish5 and #Cryptofish7 at the actin locus, the presence of two major clades was confirmed. In addition, when possible, longer 18S amplicons were generated. In conclusion, the small genetic distances between these genotypes designated as a novel marine genotype I (#Cryptofish 1-5) suggest that they may be genetic variants of the same species, while the designated novel marine genotype 2 (#Cryptofish 7) is clearly representative of a separate species.

## 1. Introduction

The protozoan parasite *Cryptosporidium* is a waterborne and foodborne pathogen, causing severe diarrhea mainly in young children and immunocompromised persons and is also found in a wide range of vertebrate hosts [1].

To date, *Cryptosporidium* spp. have also been genetically characterized in more than 25 species of both freshwater and marine fish [2]. Four species and more than 20 piscine genotypes, all with strong host specificity and no reports in humans have been identified, including *Cryptosporidium molnari* [3,4], *C. scophthalmi* [5], *C. huwi* (previously known as piscine genotype (1) [6] and *C. bollandi* (previously known as piscine genotype (2) [7,8], piscine genotypes 3–8 [9,10,11,12], piscine genotype 9 [13], a *C. molnari*-like genotype [2,10,14,15,16,17], five unnamed novel genotypes [15,16], a Koi-Carp genotype [18] and Cryptofish 1–5 and 7 [2]. In addition, other *Cryptosporidium* spp. that are commonly identified in mammals, such as *C. parvum*, *C. hominis*, *C. scrofarum*, *C. xiaoi* [2,9,12,13,19] and *Cryptosporidium* rat genotype III-like genotype have also been found in fish [11].

Molecular studies previously conducted on fish species revealed an extensive genetic diversity of *Cryptosporidium* spp. isolates [15,16]. However, the majority of studies on piscine *Cryptosporidium* have been carried out on ornamental or farmed fish, and scarce data are currently available concerning the molecular identification of *Cryptosporidium* species and genotypes in wild marine fish.

In a recent work evaluating the prevalence of *Cryptosporidium* spp. in commercially important edible marine fish in different European seas, sequence and phylogenetic analysis at the 18S rDNA locus identified 6 novel genotypes of *Cryptosporidium* colonizing these hosts [2]. Of these six genotypes, five grouped together (#Cryptofish 1–5) whereas #Cryptofish 7 emerged separately [2]. Based on the molecular characteristics of these genotypes, the main goal of the present study was thus to perform a comparative genetic characterization at the actin locus between these novel genotypes and with available piscine-derived Cryptosporidium genotypes, to provide further evidence for them as potential new separate species. A more comprehensive Cryptosporidium taxonomy is of major interest for a better understanding of transmission dynamics, public health significance and biology of this parasite.

## 2. Materials and Methods

### 2.1. Sampling

Scrapings of the gastrointestinal epithelia from several edible marine fish species (*Merlangius merlangus*, *Pollachius virens*, *Molva dypterygia* and *Scomber scombrus*) caught in different European seas (English Channel, North Sea, Bay of Biscay, Celtic Sea and Mediterranean Sea) that were previously identified as positive for different *Cryptosporidium* genotypes at the 18S rDNA locus [2] were selected. Samples were preserved in RCL2^®^ buffer (Alphelys, Plaisir, France) and stored at −20 °C until required. Sections of the stomach and/or intestine were also collected and fixed in 10% buffered formalin for further histological analysis (Table 1). 

The fish species were collected through research cruises belonging to the French Institut Français de Recherche pour l’Exploitation de la Mer (Institut Français de Recherche pour l'Exploitation de la Mer-IFREMER) in different European seas or through purchases from wholesalers or retailers for commercial catches at Boulogne-sur-Mer (Global Positioning System Coordinates: 50°43′ N–1°37′ E), the first French fishing port [2,20]. Ifremer research cruises are carried out with the French Oceanographic Fleet under the supervision of the French Ministry of Education and Research. A steering committee evaluates and approves the entire scientific campaign program before implementation. The study was performed in accordance with the EU directive 2010/63/EU and followed all the guidelines of the deontology charter of Ifremer’s research.

### 2.2. DNA Extraction and PCR Amplification

Genomic DNA (gDNA) was newly extracted from gastric and/or intestinal scrapings of epithelia of fish, using the NucleoSpin™ Kit (Macherey-Nagel, GmbH & Co KG, Düren, Germany) as previously described [19]. DNA was diluted in 100 μL of elution buffer.

DNA from fish samples were reamplified for identification of *Cryptosporidium* at the 18S rDNA locus (~588 bp) as previously described [19]. In the present study, and when possible, longer 18S amplicons (~825 bp) were also generated using primers described by Xiao et al. [21]. Positive isolates were also analyzed at the actin locus using genus-specific actin primers described by Sulaiman et al. [22] producing a ~1066 bp amplicon. No-template controls (NTCs) were included alongside each PCR. Secondary PCR products were visualized on a 1% agarose gel stained with SYBR Safe (Thermo Fisher Scientific, Perth, Australia) fluoresce under ultraviolet light.

### 2.3. DNA Sequencing and Analysis

To identify *Cryptosporidium* species/genotypes, secondary PCR products were purified using the NucleoFast^®^ 96 PCR kit (Macherey Nagel, GmbH & Co KG, Düren, Germany). Purified PCR products were sequenced in both directions, using the secondary PCR primers (Genoscreen, Pasteur Institute of Lille, Lille, France). Obtained nucleotide sequences were aligned using the BioEdit v. 7.0.1 package, and compared with available DNA sequences of *Cryptosporidium* in GenBank data base using the NCBI BLAST basic local alignment search tool (http://www.ncbi.nlm.nih.gov/BLAST/).

For longer 18S rDNA and actin amplicons, secondary PCR products were purified using a filter tip method [23] and sequenced in both directions using an ABI Prism™ Dye Terminator Cycle Sequencing kit (Applied Biosystems, Foster City, CA, USA) according to the manufacturer’s instructions at 58 °C. Nucleotide sequences identified in this study were deposited in GenBank under the accession numbers: MT570027—MT570035 (actin locus) and MT776545—MT776547 (18S rDNA locus).

### 2.4. Phylogenetic Analysis at the Actin Locus

Sanger sequencing chromatogram files were imported into Geneious Pro 10.2.6 [24], analysed and aligned with reference sequences from GenBank (*C. huwi* (AY524772), *C. molnari* (HM365220), *C. molnari* (HM365219), *C. bollandi* (MT160193) and *C. scopthtalmi* (KR340589)) using Clustal W (http://www.clustalw.genome.jp). Distance and Maximum Likelihood (ML) trees were constructed using MEGA v. 7, after first identification of the most appropriate nucleotide substitution model [25]. Bootstrap support based on 1000 replications was included.

### 2.5. Histopathological Examination

Paraffin-embedded tissues were cut to a thickness of 5 μm and stained with hematoxylin and eosin (H & E). A DMRB microscope (Leica Wetzlar, Germany) equipped with a Leica digital camera connected to an Imaging Research MCID analysis system (MCID Software, Cambridge, UK) was used for observation of the histological sections.

### 2.6. Detection of Cryptosporidium by Immunofluorescence

A conjugated anti-*Cryptosporidium* spp. (Sporoglo, Waterborne, New Orleans, LA, USA) was used in direct fluorescent-antibody staining assay according to the manufacturers’ instructions. DAPI (4′,6-diamidino-2-phenylindole) was used for nuclei identification. Slides were examined in a LSM880 Confocal Microscope (Zeiss, Oberkochen, Germany) equiped with a UV laser for the detection of DAPI (excitation at 355 nm and emission at 405 nm) and VIS (visible light) laser for the detection of sporoglo (excitation wavelength at 555 nm and emission at 561 nm). The images were taken using Ziess Axiocam digital camera. The images were processed using Carl Zeiss Zen software.

## 3. Results

Nested 18S rDNA PCR and sequencing of DNA from piscine-derived Cryptosporidium isolates using the original shorter amplicons products previously described [2] confirmed their assignment to different genotypes as follows: nine samples belonged to genotype #Cryptofish1 (MK236538), one to genotype #Cryptofish2 (MK236539), another one to #Cryptofish4 (MK236541), two corresponded to genotype #Cryptofish5 (MK236542) and one to genotype #Cryptofish7 (MK236544). However, longer 18S amplicons (~825bp) were only generated successfully for genotype #Cryptofish1 (MT776545, MT776546 and MT776547).

**Figure 1:**
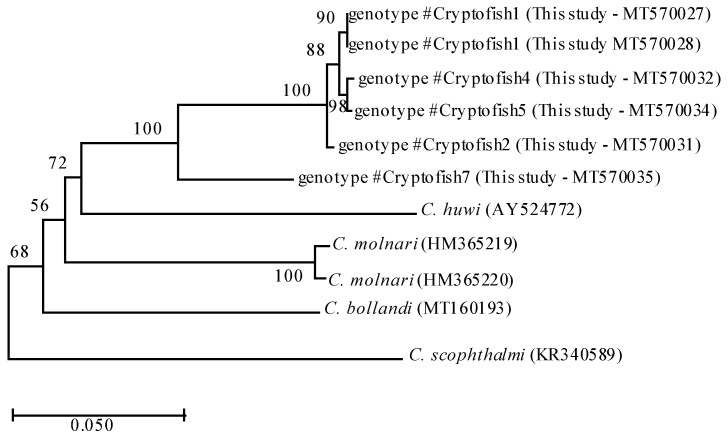
Phylogenetic relationships between #Cryptofish1, 2, 4, 5 and 7 genotypes (identified in the present study) and other piscine *Cryptosporidium* species inferred by ML analysis of actin gene. Percentage support (>50%) from 1000 pseudoreplicates from ML analyses is indicated at the left of the supported node. Scale bars indicate the number of substitutions per nucleotide position.

Actin sequences obtained for six samples were representative of #Cryptofish1, #Cryptofish2, #Cryptofish4, #Cryptofish5 and #Cryptofish7 (Table 1). Phylogenetic analyses of #Cryptofish1, #Cryptofish2, #Cryptofish4, #Cryptofish5 and #Cryptofish7 at the actin locus identified two major clades: one composed of the sequences of genotypes #Cryptofish1, #Cryptofish2, #Cryptofish4 and #Cryptofish5 and the other one composed of the sequence of #Cryptofish7 (Figure 1). At the actin locus, the #Cryptofish1 genotype exhibited 0.9% genetic distance from #Cryptofish2, 0.5–0.6% from #Cryptofish4 and #Cryptofish5 and 9.1% from #Cryptofish7, while #Cryptofish2 exhibited 8.2% genetic distance from #Cryptofish7. Finally, #Cryptofish7 exhibited 14.3%, 15.5%, 16.2% and 20.2% genetic distance from *C. molnari*, *C. bollandi*, *C. huwi*, and *C. scophtalmi*, respectively (Figure 1 and Table 2). The small genetic distances at the actin locus suggests that #Cryptofish1, #Cryptofish2, #Cryptofish4 and #Cryptofish5 (designated novel marine genotype 1) may be genetic variants of the same species, while #Cryptofish7 (designated novel marine genotype 2) is clearly a separate species (Table 2).

**Figure 2:**
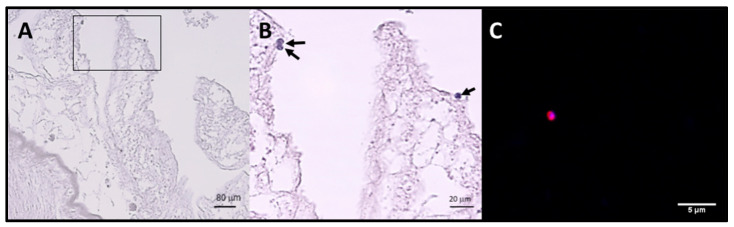
Stained sections of the intestinal tract of one *Scomber scombrus* (Ss VII 4BO) infected by the genotype Cryptofish7 (**A**) Presence of round bodies suggestive of the developmental stages of *Cryptosporidium* spp. observed in the apical position of a (stained with H&E) (**B**) Detail of A (delimited area). (**C**) Structure labelled in red with the Sporoglo (Waterborne) antibody and blue with DAPI (4′,6-diamidino-2-phenylindole) suggestive of an intracellular stage of *Cryptosporidium* spp.

After examination of histological sections from the digestive tract of fishes, the presence of *Cryptosporidium*-like bodies in apical position was observed (Figure 2A,B). These structures were 3–4 μm in diameter, spherical and positive in H&E. Immunofluorescence analysis confirmed this with structures similar in size and shape to intracellular stages of Cryptosporidium labelled in red with the Sporoglo antibody (Figure 2C). However, the presence of parasites could not be confirmed in all positive samples due to substantial lysis of tissues.

## 4. Discussion

Molecular characterization of *Cryptosporidium* has contributed to a better understanding of the diversity and transmission dynamics of this important enteric parasite. In the present study, we analyzed new genotypes of Cryptosporidium previously identified in edible fish [2] at the actin locus in order to provide additional data to support their potential species status.

Previous sequence and phylogenetic analysis at the 18S rDNA gene locus had shown that these genotypes were distributed as follows: 22 (48%) belonged to the #Cryptofish1 genotype that exhibited 7.3–8.5% genetic distance from *C. molnari*, six (13%) belonged to another genotype #Cryptofish2 that exhibited 8.5–9.5% genetic distance from *C. molnari*, a single isolate (2%) identified as #Cryptofish4 exhibited 8.2–9.5% genetic distance from *C. molnari,* 4 (9%) belonged to #Cryptofish5 genotype, which exhibited 7.6–8.8% genetic distance from *C. molnari*. Finally, the single isolate (2%) #Cryptofish7 exhibited 9.1–10.4% genetic distance from *C. molnari* [2]. #Cryptofish3 genotype which was previously identified [2] and which exhibited 8.9–10.1% genetic distance from *C. molnari* could not be analyzed in the current study.

In the previous analyses at the 18S rDNA locus, the genetic distances between #Cryptofish1, #Cryptofish2, #Cryptofish4 and #Cryptofish5 was 0.3–2.8% and these sequences exhibited 6.1–8.9% genetic distances from #Cryptofish7. Interestingly, in the present study, at the actin locus, the genetic distances between #Cryptofish1, #Cryptofish2, #Cryptofish4 and #Cryptofish5 were smaller (0.6–0.9%) and they exhibited 8.2–9.1% genetic distances from #Cryptofish7. #Cryptofish1, #Cryptofish2, #Cryptofish4 and #Cryptofish5 exhibited 14.0–14.5% (#Cryptofish2 and *C. molnari*) to 19.5% (#Cryptofish4 and *C. scophtalmi*) genetic distance from other fish species. #Cryptofish7 exhibited 14.3%, 15.5%, 16.2% and 20.2% genetic distance from *C. molnari*, *C. bollandi*, *C. huwi*, and *C. scophtalmi*, respectively (Table 2).

The genetic distances between #Cryptofish1, #Cryptofish2, #Cryptofish4 and #Cryptofish5 may be enough to suggest that they are separate species. For example, the genetic distance at both the 18S rDNA and actin loci between *C. erinacei* and *C. parvum* is 0.5% [26] and the genetic distance between *C. muris* and *C. andersoni* at the 18S rDNA and actin loci is 0.9% and 3.5%, respectively. However, further analyses are required to confirm this and they are currently grouped together as novel marine *Cryptosporidium* genotype 1. Additional biological characteristics of the two potential novel marine genotypes are listed in Table 3.

This genotype was the most prevalent since it was identified either in the stomach or the intestine of five different fish species belonging to the order gadiformes, all caught in the Atlantic North East (*Pollachius virens, Molva dysterygia, Molva molva, Merlangius merlangus* and *Merlucius merlucius*, with *P. virens* as the most common fish host). The second novel species (#Cryptofish7) (novel marine genotype 2) was identified in the stomach of a single specimen from *Scomber scombrus* (order scombriformes) caught in the English Channel (Table 3) [2]. Interestingly, it has been described that genetically related hosts often harbor related species of *Cryptosporidium* [27]. However, further studies are needed to confirm any specificity related to fish orders, considering that #Cryptofish7 was only found in a single specimen.

In order to verify multiplication of parasites in fish digestive tissues, histological sections from the digestive tract of fishes were analyzed after staining with H&E or by immunofluorescence analysis. Cryptosporidium-like bodies were observed in an apical position suggesting the multiplication of the parasite and a true infection of the fish rather than just carriage (Figure 2). In addition, as DNA screening was conducted on mucosal scrapings from intestines and stomach tissue, this also suggests actual infections. The presence of parasites could not be studied in all positive fishes due to considerable lysis of tissues, which has been previously described as a difficulty for *Cryptosporidium* detection in fish hosts [10,11,12]. In addition, tissues were not available for all specimens. Unfortunately, oocysts could not be obtained to determine the morphological features of the oocyst stage of these piscine genotypes. However, morphological overlap in oocyst size is commonly found among *Cryptosporidium* spp., and in particular among *Cryptosporidium* spp. from fish [6]. Moreover, it is widely recognized that morphometrics is not a useful tool for defining most species within this genus [28].

The pathogenesis of the *Cryptosporidium* species identified in the present study remains unknown. Nevertheless, different studies have reported that piscine species and genotypes can cause pathological effects in fish [3,5] as well as an increase in mortality, particularly in juveniles [8], negatively impacting the fish industry economy.

The high diversity of *Cryptosporidium* species and genotypes in fish indicates a long-term association of *Cryptosporidium* with their fish hosts. The understanding of the taxonomy of piscine-derived *Cryptosporidium* species is of relevance considering that previous studies suggest that they might represent the most primitive *Cryptosporidium* species 6 [10].

Cryptosporidiosis can be considered important in fish since these animals may act as carriers and may be a source of infection for other hosts including humans. Further studies should be conducted to determine if contact with edible marine fish colonized by these Cryptosporidium species/genotypes pose a risk of zoonotic transmission either through their consumption and/or handling or through the consumption of water contaminated with fully sporulated oocysts shed in fish feces [29]. The four already known piscine species of *Cryptosporidium* have not yet been found in other hosts [1,30,31,32] suggesting that these species may not be able to grow in the digestive tract of mammals. However, further research is required to confirm this hypothesis.

## 5. Conclusions

In the present study, two potential novel *Cryptosporidium* fish species were further characterized at the actin locus. Further investigations will be performed in order to confirm that these genotypes are different *Cryptosporidium* species.

*Cryptosporidium* spp. have a wide host range and this together with the potential for high levels of oocyst shedding, allows a significant level of contamination of the environment. In particular, for fish hosts, the dispersion and transmission of zoonotic parasites would be facilitated by the aquatic habitat of the host, that could potentially release oocysts contributing to Cryptosporidium circulation. Therefore, additional epidemiological studies in wildlife animals are needed to better define the host range and zoonotic potential of the parasite.

## Figures and Tables

**Table 1:** Details of *Cryptosporidium* piscine isolates typed at the 18S locus in a previous study and re-amplified and sequenced at the 18S rDNA and actin loci for the present study.

Sample Identification	Fishing Area	Fish Scientific Name	Fish Common Name	Order	Organ Location	Genotype Identified (18S rDNA )	GenBank Accession Numbers (18S rDNA)	GenBank Accession Numbers (Actin)
524	NE ^a^ Atlantic	*M. merlangus*	Whiting	Gadiformes	Stomach	Cryptofish2	MK236539 and MT776545	MT570031
698	NE Atlantic	*P. virens*	Saithe	Gadiformes	Stomach	Cryptofish1	MK236538	NA ^b^
710	NE Atlantic	*P. virens*	Saithe	Gadiformes	Stomach	Cryptofish1	MK236538 and MT776546	MT570028
716	NE Atlantic	*P. virens*	Saithe	Gadiformes	Stomach	Cryptofish1	MK236538	NA
719	NE Atlantic	*P. virens*	Saithe	Gadiformes	Intestine	Cryptofish1	MK236538	NA
720	NE Atlantic	*P. virens*	Saithe	Gadiformes	Stomach	Cryptofish1	MK236538	NA
722	NE Atlantic	*P. virens*	Saithe	Gadiformes	Intestine	Cryptofish1	MK236538 and MT776547	MT570027
735	NE Atlantic	*M. dypterygia*	Blue ling	Gadiformes	Stomach	Cryptofish4	MK236541	MT570032
PV-III-4 BO	NN ^c^ Sea	*P. virens*	Saithe	Gadiformes	Intestine	Cryptofish1	MK236538	MT570030
PV-IV-3 ST	NN Sea	*P. virens*	Saithe	Gadiformes	Stomach	Cryptofish1	MK236538	MT570029
PV-IV-2 BO	NN Sea	*P. virens*	Saithe	Gadiformes	Intestine	Cryptofish1	MK236538	NA
PV-IV-7 ST	NN Sea	*P. virens*	Saithe	Gadiformes	Stomach	Cryptofish5	MK236542	MT570034
PV-IV-10 ST	NN Sea	*P. virens*	Saithe	Gadiformes	Stomach	Cryptofish5	MK236542	MT570033
SS-VII-4 ST	English Channel	*S. scombrus*	Mackerel	Scombriformes	Stomach	Cryptofish7	MK23654	MT570035

^a^ NE: North East, ^b^ NA: Not available ^c^ NN: Northern North.

**Table 2:** Pairwise genetic distances (%) between the piscine *Cryptosporidium* spp. and genotypes found in the present study at the actin locus based on 722 bp sequences.

Piscine *Cryptosporidium*	#CryptoFish1	#CryptoFish2	#CryptoFish4	#CryptoFish5	#CryptoFish7	*C. huwi*	*C. molnari*	*C. molnari*	*C. bollandi *
#CryptoFish1 (Intestine, MT570028)	-	-	-	-	-	-	-	-	-
#CryptoFish2 (MT570031)	0.9	-	-	-	-	-	-	-	-
#CryptoFish4 (MT570032)	0.5	0.8	-	-	-	-	-	-	-
#CryptoFish5 (MT570034)	0.6	0.7	0.004	-	-	-	-	-	-
#CryptoFish7 (MT570035)	9.1	8.2	9.2	8.8	-	-	-	-	-
*C. huwi* (AY524772)	16.5	16.1	16.6	16.4	16.2	-	-	-	-
*C. molnari* (HM365220)	15.1	14.5	15.2	15.1	14.3	17.7	-	-	-
*C. molnari* (HM365219)	14.5	14.0	14.7	14.5	14.3	18.4	0.7	-	-
*C. bollandi* (MT160193)	17.1	16.5	17.2	17.1	15.5	18.4	15.5	15.7	-
*C. scopthtalmi* (KR340589)	19.0	18.6	19.5	19.3	20.2	24.3	17.6	17.4	20.2

**Table 3:** Characteristics of novel piscine *Cryptosporidium* genotypes identified in the present study.

Novel Marine Genotypes	Genotypes	Fish Host Scientific Name (Fish Host Common Name) ^a^	Order	Fishing Area ^a^	Organ Distribution ^a^	Overall Prevalence in Fish ** %** (*n* = 1853) ^a^	Distribution According to *Cryptosporidium* Positive Cases % (*n* = 46) ^a^
Novel marine genotype 1	#Crypto1, #Crypto2, #Crypto4, and #Crypto5	*P. virens* *(Saithe)* *Molva dypterygia* *(Blue ling)* *Molva molva* *(Ling),* *Merlangius merlangus* *(Whiting)* *Merluccius merluccius* (Hake) *	Gadiformes	NE Atlantic	Intestine and/or stomach	1.78	71.7
Novel marine genotype 2	#Cryptofish7	*Scomber scombrus (Mackerel)*	Scombriformes	English channel	Stomach	0.05	2.1

^a^ Source [2]. * This fish species was found positive for this novel marine genotype in a previous study [2] but unfortunately, there was insufficient sample to reamplify and sequence again.
